# The introduction of a video corner in Facts, View & Vision: why and when?

**Published:** 2017-06

**Authors:** Vasilis Tanos, Rudi Campo, Willem Ombelet

**Affiliations:** Aretaeio Hospital and St Georges Medical School, Nicosia University, Nicosia, Cyprus.; Genk Institute for Fertility Technology, 3600 Genk, Belgium; Life Expert Centre, Schipvaartstraat 4, 3000 Leuven, Belgium; Editor in Chief

The idea of a Video Corner in our Journal has been discussed in the last 2 years, inspired by numerous video sessions introduced by many ObGyn societies during their meetings. Technological advances matured and provide today uninterrupted and high clarity videos. Based on the moto that one picture is worth thousand words you can imagine the impact on teaching ability of a short video when a new operative technique or the management of an obstetrical complication and its treatment can be presented.

This Journal embraces all ObGyn disciplines from the beginning and it always showed a major interest in images, art contributions and visionary ideas. Therefore we believe that it provides the perfect scientific environment and conditions to initiate and support the first of its kind video journal in medical profession.

Traditional educational methods and presentation of academic work by written articles definitely have a great value, perspective and of course limitations. Our Video Corner aims to facilitate further learning and understanding, especially when complex matters are involved. High definition audiovideo presentations, accompanied by clear and evidence based articles or case-reports provide, in our opinion, the best option to show and explain complicated cases and pathologies that normally need extensive writing to describe and fully understand the case. Within this context a video corner with ObGyn articles can improve educational skills and shorten learning curves.

In addition, important details of an operation, rare pathologies, anatomical deviations and surgeon ergonomy can be demonstrated in a better way. Many operative procedures are poorly described, due to the limitations inherent to written papers. Hence, video articles have an added value in learning difficult tasks and open a new way to transfer medical information and knowledge which is not possible without a video presentation. We are also open for new techniques and innovative methods, setting up unusual projects etc., as long as the guidelines are followed. Video articles presenting the management of complications during surgical and medical treatments are urgently needed. It is of imperative value since concerns patients’ safety and surgery outcome.

It is our aim that the guidelines to authors considering abstract, conclusions and references remain the same as for the written format articles in our journal. However, the paper should be presented with restricted word numbers and accompanied with video sessions. It will be mandatory to present in each video article the learning objectives in a constructive and clear way and to discuss crucial scientific matters and conclusive remarks.

The video articles will be reviewed by a group of experts of each discipline in ObGyn. The important factors to accept a video article is the video content fulfilling the written learning tasks and the importance of the presenting objectives.

In the next issue of FV&V we will present the editorial board, the guidelines to follow and an example of a video article. From November on it will be possible to submit your contributions to the Journal.

